# Sphingosine Kinase 2 Phosphorylation of FTY720 is Unnecessary for Prevention of Light-Induced Retinal Damage

**DOI:** 10.1038/s41598-019-44047-z

**Published:** 2019-05-23

**Authors:** Hui Qi, Jerome Cole, Richard C. Grambergs, John R. Gillenwater, Koushik Mondal, Sufiya Khanam, Soma Dutta, Megan Stiles, Richard L. Proia, Jeremy Allegood, Nawajes Mandal

**Affiliations:** 1 Department of Ophthalmology, University of Oklahoma Health Sciences Center (OUHSC), Oklahoma City, OK 73104 USA; 2 Department of Ophthalmology, University of Tennessee Health Science Center, Memphis, TN 38163 USA; 3 Genetics of Development and Disease Branch, National Institute of Diabetes and Digestive and Kidney Diseases, National Institutes of Health, Bethesda, MD 20892 USA; 4 Department of Biochemistry and Molecular Biology, Virginia Commonwealth University School of Medicine, Richmond, VA 2329 USA; 5 Department of Anatomy and Neurobiology, University of Tennessee Health Science Center, Memphis, TN 38163 USA

**Keywords:** Sphingolipids, Molecular medicine, Translational research

## Abstract

Mammalian Sphingosine kinase 2 is the primary enzyme responsible for phosphorylating FTY720 to its active form, FTY720-P. Systemic FTY720 treatment confers significant protection to murine retinas from light- and disease-mediated photoreceptor cell death. It is not clear whether FTY720-P, FTY720, or both are responsible for this photoreceptor protection. We investigated *Sphingosine kinase* 2 knockout (*Sphk2* KO) mouse retinas, tested their sensitivity to light, and measured what degree of protection from light-induced damage they receive from systemic FTY720 treatment. *Sphk2* KO retinas were found to be similar to their wild-type counterparts in sensitivity to light damage. Additionally, FTY720 treatment protected *Sphk2* KO retinas from light-induced damage despite significant retardation of FTY720 phosphorylation in *Sphk2* KO mice. We conclude that FTY720 serves an active role in preventing photoreceptor cell death. Furthermore, we conclude that the phosphorylation of FTY720 is not necessary to provide this protective effect.

## Introduction

FTY720 is an FDA-approved human drug for relapsing multiple sclerosis (MS) under the trade name Gilenya. In MS, FTY720 has immunosuppressive qualities and acts on immune cells at lymph nodes following conversion to FTY720-phosphate (FTY720-P) in the liver^1–4^. FTY720 is a synthetic derivative of the fungal toxin myriocin and is a structural analog of the cellular bioactive sphingolipid sphingosine (Sph). The major source of Sph in cells is via the breakdown of ceramide (Cer), another bioactive sphingolipid and a potent signaling molecule for inflammation and cell death. Sph can be converted back to Cer through a salvage pathway, or can serve as substrate for two kinases, Sphingosine kinase 1 (SPHK1) and SPHK2, to generate another important pleiotropic signaling sphingolipid, sphingosine 1-phosphate (S1P). S1P mediates multiple intracellular functions important for cellular survival, essentially acting in opposition to Cer. S1P also serves as a ligand for a family of five known G-protein coupled receptors, S1PR1–5, which are present on endothelial cells, neuronal cells, and immune cells, and modulate cell migration, cell adhesion, inflammation, and new vessel formation^5–14^.

Being an analog of Sph, FTY720 is rapidly phosphorylated to FTY720-P by both SPHK1 and SPHK2, at which point the phosphorylated form mimics S1P. This conversion primarily occurs in the liver via SPHK2^15–19^. Genomic analysis reports that expression of SPHK1 is highest among lung and spleen tissue while SPHK2 is preferentially expressed in the heart and liver^16,20,21^. Although the two enzymes’ conserved domains share 80% homology, they exhibit distinct kinetic differences: SPHK2 is quantitatively 30-fold more efficient at phosphorylating FTY720 compared to SPHK1 due to the drug’s lower Km towards the second isotype^15,18,19^. Thus, most FTY720 is converted to FTY720-P in the liver by SPHK2.

We have shown previously that systemic dosing of FTY720 before exposure to intense, damaging light provides significant protection to photoreceptor cells from apoptotic cell death^22,23^. From the kinetics of FTY720-mediated prevention of *in vivo* photoreceptor cell death in animal models, it appears that the unphosphorylated form of FTY720 provides sufficient protection from apoptotic cascades^22^. FTY720 treatment completely blocked intense light exposure-induced ceramide increases prior to cell death. We found that Cer plays a crucial role in light-induced degeneration of photoreceptor cells and were able to conclude that FTY720 prevents excess Cer formation during light exposure^22^. We have also shown in our *in vitro* studies that reduction of Cer by overexpression of acid Ceramidase (ASAH1), a lysosomal enzyme which degrades excess Cer, in retinal pigment epithelial (RPE) cells provides significant protection from oxidative stress-induced cell death, further highlighting the role of Cer in retinal degeneration^24^.

Retinal photoreceptor cell death by apoptosis is the hallmark of major retinal degenerative diseases such as retinitis pigmentosa (RP), and is also a major component of diabetic retinopathy (DR) and age-related macular degeneration (AMD)^25–27^. So far, no effective therapies have been developed for this group of diseases. Our findings that FDA-approved FTY720 plays an active, protective role in the prevention of retinal cell death opened the door to many potential avenues for further investigation of the complex pathophysiology of human retinal degenerative diseases and potential therapy development. However, it is important to understand the actual mechanisms of FTY720 in retinal protection, as it can act as a pleiotropic molecule in addition to its known effect of inhibiting *de novo* Cer synthesis^28,29^. As a mimic of bioactive S1P, FTY720-P can have a multitude of actions and signaling roles in mammalian systems that may directly or indirectly affect the fate of photoreceptors during light exposure. The current understanding of the mechanisms driving these effects is far from complete and must be further developed. To that end, we designed the present study to test whether it is FTY720 or FTY720-P that is ultimately responsible for previously-observed photoreceptor protection in light-stressed murine models. We converted *Sphk2* knockout (KO) mice (which are severely deficient in their ability to phosphorylate FTY720 to FTY720-P) to an albino background (which is vulnerable to light-induced retinal damage) and administered FTY720 intraperitoneally prior to subjecting them to a period of intense light stress designed to model photoreceptor damage. Our experiments provided convincing evidence that FTY720 itself can provide protection from light-induced damage to retinal cells without conversion to FTY720-P.

## Results

### Generation of albino *Sphk2 KO* mice

We received *Sphk2* KO mice on C57BL/6 background from Dr. Richard L. Proia (NIDDK, Bethesda, MD)^13^. As pigmented mice are resistant to light-induced retinal degeneration and unsuitable for our model, we generated an albino *Sphk2* KO line by 7-generation backcrossing with Balbc mice. After genotyping, heterozygous females were selected from each generation and backcrossed to a parent Balbc male. Like the parent line, the albino line is also viable and fertile^13^.

### Visual characterization of *Sphk2 KO* mice

**Figure 1:**
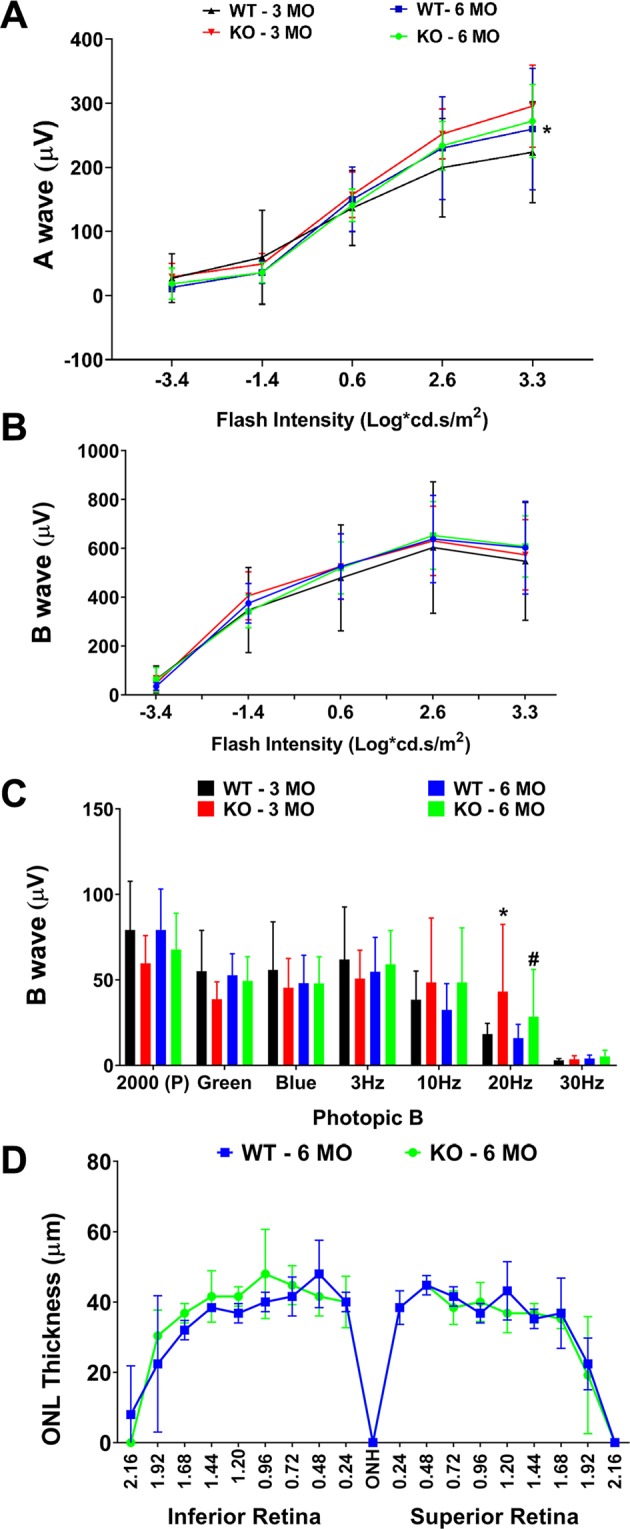
Functional and Structural characterization of *Sphk2* knockout mouse retinas. (**A**) Rod photoreceptor function measured by dark-adapted scotopic ERG – A wave amplitudes (µV) at 3 and 6 months of age at increasing flash intensities (0.0004–2000 cd.s/m^2^) and presented in line graph with mean ± S.D. (**B**) Scotopic ERG B wave amplitudes (µV) at 3 and 6 months of age at increasing flash intensities (0.0004–2000 cd.s/m^2^) are presented in line graph with mean ± S.D. (**C**) Cone photoreceptor function measured by ERG at 3 and 6 months of age and photopic B wave amplitudes (µV) are presented in bar graph with mean ± S.D. (**D**) Outer Nuclear Layer (ONL) thickness measurements are presented in line graph with mean ± S.D. WT, wild-type; KO, *Sphk2* knockout; MO, Month; *p < 0.05; ^#^p < 0.05. *Represents significant difference between WT and KO mice in the 3-month group; ^#^represents significant difference between WT and KO mice in the 6-month group.

We conducted basic visual functional characterization of albino *Sphk2* KO and wild-type (WT) mice by measuring retinal function using ERG at 3 and 6 months of age. We also analyzed retinal/eye structure with histology. We did not detect any major changes in scotopic ERG A-wave and B-wave (a measure of rod photoreceptor function), aside from a measurable difference in A-wave at the highest intensity flash (2000 cd.s/m^2^) between the WT and KO mice at 3 months of age (Fig. 1A,B; p < 0.05, n = 12; t-test). Using photopic ERG measuring cone photoreceptor function, no difference was observed with bright white (2000P), green, and blue light. The KO animals (both 3 and 6 months old) showed higher response at 20 Hz flickers, which measure cone photopigment regeneration (Fig. 1C; p < 0.05, n = 12 (3-Mo), n = 10 (6-Mo); t-test). ERG representative traces at different flash intensities of WT and KO mice at 3 and 6 months indicated similar photoreceptor viability between WT and *Sphk2* KO mice, suggesting that knockout of the *Sphk2* gene does not cause any apparent defects in photoreceptor performance (Supplementary Fig. S1). Histological analysis did not reveal any distinct differences in retinal structure and organization of various layers between KO and WT mice. As expected from the photoreceptor function study, there was no difference in the thickness of the photoreceptor layer in KO mice at 6 months of age (Fig. 1D; n = 4).

### Light damage response of *Sphk2 KO* mice retina and the effect of FTY720

**Figure 2:**
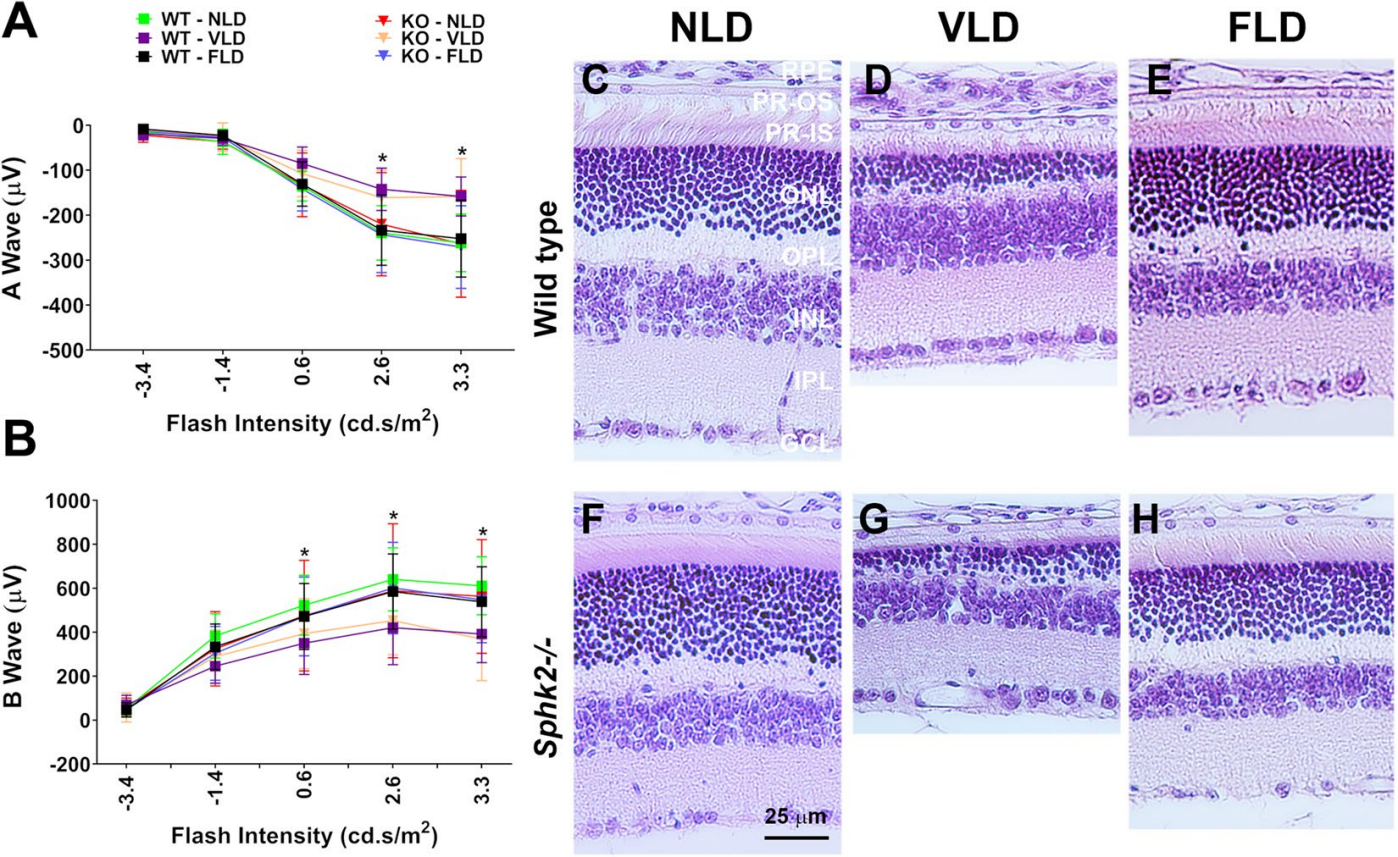
Characterization of *Sphk2* knockout mouse retina after Light Induced Retinal Damage. Seven days after light-induced retinal damage (LD), photoreceptor function of WT and *Sphk2* KO mice injected with either FTY720 or Vehicle was measured using ERG. (**A**) Rod photoreceptor function measured by dark-adapted scotopic ERG A wave amplitudes (µV) and presented in line graph representing mean ± S.D. (**B**) Scotopic ERG B wave amplitudes (µV) at 3 and 6 months of age at increasing flash intensities (0.0004–2000 cd.s/m^2^) are presented in line graph with mean ± S.D. (**C**–**H**) Representative H&E-stained sections of both wild-type and knockout mice retinas were imaged from the superior central retina: (**C**) wild-type no light damage (NLD); (**D**) wild-type vehicle-injected and light damage (VLD); (**E**) wild-type FTY720-treated and light damage (FLD); (**F**) knockout NLD; (**G**) knockout VLD; (**H**) knockout FLD. *p < 0.05 WT-VLD vs. WT-NLD; ^#^p < 0.05 KO-VLD vs. KO-NLD. WT, wild-type; KO, *Sphk2* knockout; NLD, No Light Damage; VLD, Vehicle + Light Damage; FLD, FTY720 + Light Damage; RPE, retinal pigmented epithelium; PR-OS, photoreceptor outer segment; PR-IS, photoreceptor inner segment; ONL, outer nuclear layer; OPL, outer plexiform layer; INL, inner nuclear layer; IPL, inner plexiform layer; GCL, ganglion cell layer.

**Figure 3:**
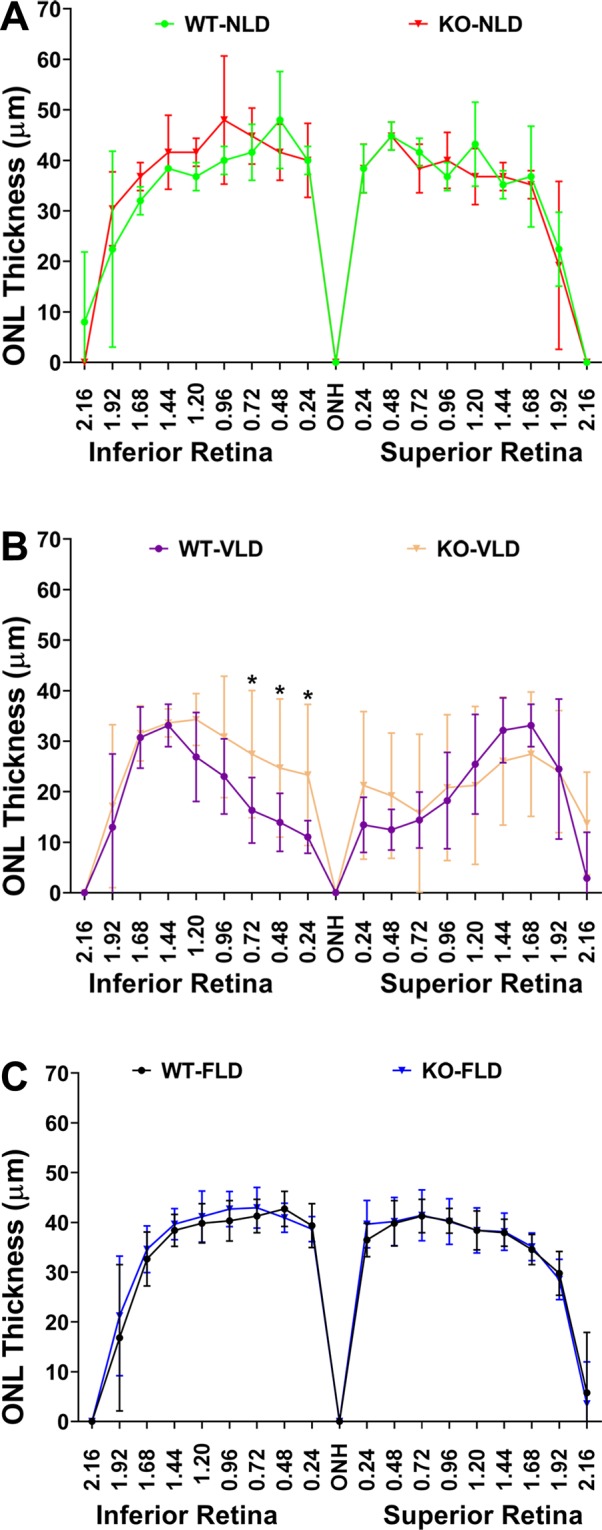
Outer nuclear layer thickness measurements of *Sphk2* knockout and wild-type mice subjected to light damage or no light damage and FTY720 intraperitoneal injection, Vehicle injection, or no injection. ONL thickness was measured along the vertical meridian from the superior to the inferior retina and presented with mean ± S.D. (**A**) WT and KO mice without light damage (NLD). (**B**) Veh-treated WT and KO mice with light damage (VLD). (**C**) FTY720-treated WT and KO mice with light damage (FLD). *p < 0.05 WT-VLD vs. KO-VLD. WT, wild-type; KO, *Sphk2* knockout.

We further characterized *Sphk2* KO mice after stressing their retinas with intense light exposure at 1000 lux for 10 hours at night when the mice are alert and active (6 PM to 4 AM). This exposure caused significant reduction in retinal function for both WT and *Sphk2* KO mice and no significant differences in retinal functional damage were found between WT and KO mice (Fig. 2A; p < 0.05, n = 6–12; two-way ANOVA with Bonferroni’s correction). However, a slight difference in structural damage was observed in the inferior retina, as shown for the vehicle-treated mice exposed to similar light conditions in Fig. 3B (p < 0.05, n = 6–12; two-way ANOVA with Bonferroni’s correction). These data indicate that *Sphk2* KO mice are almost equally susceptible to light-damage (LD) compared to WT mice.

We wanted to determine whether FTY720 or FTY720-P plays a major role in preventing retinal light damage. Following previously published protocols, 10 mg/kg FTY720 and vehicle (Veh) were delivered systemically via intraperitoneal (IP) injections to *Sphk2* KO and WT mice 30 minutes prior to LD. Both WT and KO mice treated with Veh and exposed to LD (VLD) had significantly reduced A-wave response (Fig. 2A; p < 0.05, n = 6–12; two-way ANOVA with Bonferroni’s correction). FTY720 treatment prevented reduction in A-wave response completely in both WT and KO mice (Fig. 2A). We observed no significant changes in A-wave responses at any flash intensity among animals treated with FTY720 prior to LD (FLD) from no-light-damaged (NLD) controls, regardless of *Sphk2* KO status (Fig. 2A). Similarly, no difference in B-wave response was observed between Veh-treated KO and WT, or between mice treated with FTY720 and NLD (Fig. 2B), indicating LD significantly reduced retinal function in both Veh-treated WT and KO mice. However, we noted complete protection of retinal function from LD when these mice are treated with FTY720 (Fig. 2B). When we looked at cone function by photopic ERG, we observed the same trend - FTY720 treatment prevented the loss of cone function in both KO and WT mice similarly (Supplementary Fig. S2).

In Fig. 2C–H, we show the representative images of the superior-central-retina of WT and KO mice from various treatments. This part of the murine retina is known to be the most affected by LD^30,31^. LD caused prominent damage to the photoreceptor cells, as indicated by the reduction of the photoreceptor layer (retinal outer nuclear layer, ONL) in both WT and KO mice treated with Veh (Fig. 2D,G). However, treatment with FTY720 prevented this damage (Fig. 2E,H). When we quantified the damage by morphometric measurement of the ONL, we found that VLD mice had significantly decreased ONL thickness, higher in the superior than the inferior retina. Mice injected with FTY720 prior to LD showed no significant deviation from NLD mice in ONL thickness, regardless of KO or WT genotype. The inferior ONL of vehicle-treated KO mice showed less light-induced degeneration than WT mice, but fewer differences were seen in the superior ONL (Fig. 3). Averaged measurements of ONL thickness at the superior and inferior central retinas showed that Vehicle-treated WT and KO mice subjected to LD had significant reduction of ONL thickness in both the superior and inferior retina, while NLD mice and mice treated with FTY720 had no significant changes in ONL thickness (Supplemental Fig. S3; p < 0.05; two-way ANOVA with Bonferroni’s correction). In summary, LD caused significant damage of retinal structure and function in both WT and KO mice, which is prevented by FTY720 injections prior to LD.

### Sphingolipid profile in *Sphk2 KO* mice tissues

**Figure 4:**
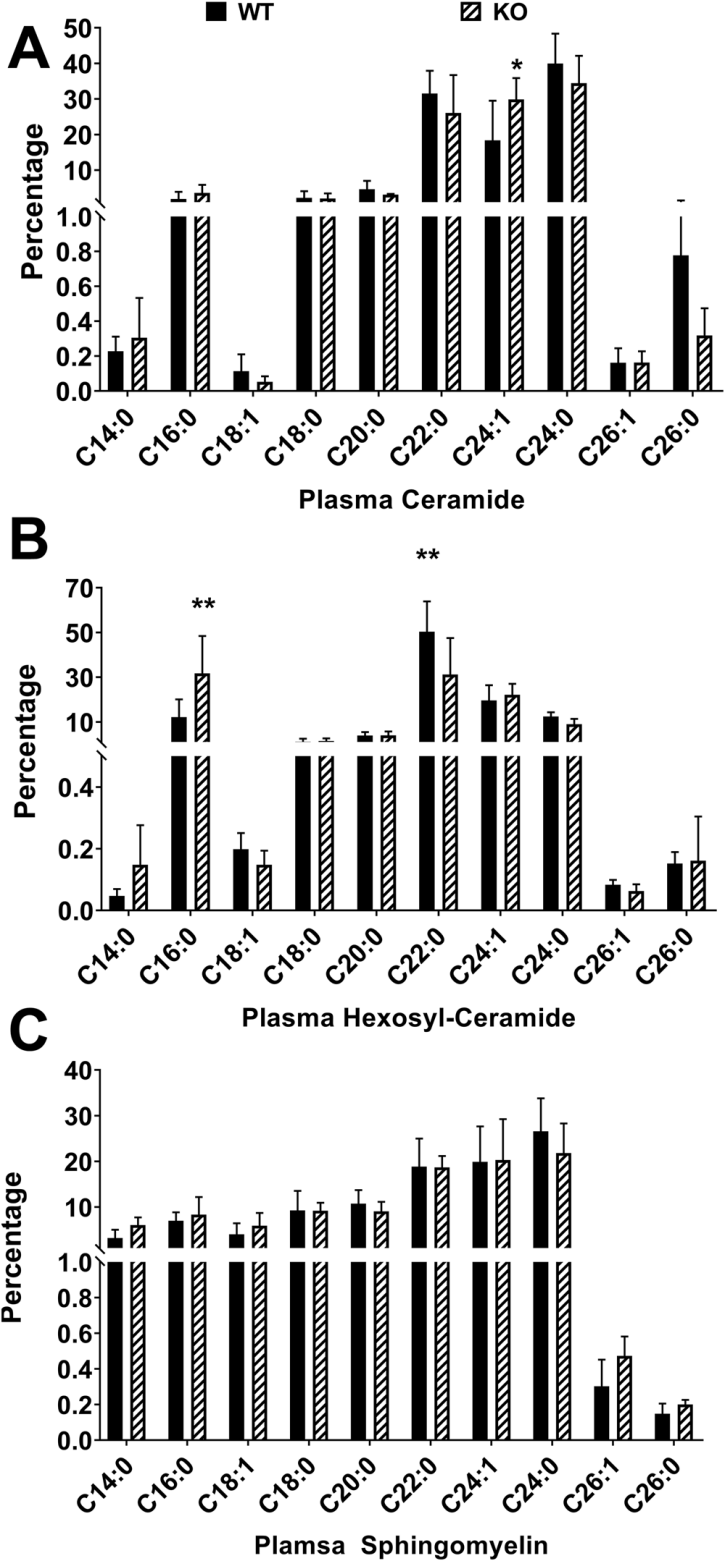
Sphingolipid Species of *Sphk2* knockout mice in plasma samples. Plasma samples were collected from WT and *Sphk2* KO mice and analyzed using LC/MS/MS for relative levels of various chain length variants of the major sphingolipid classes: (**A**) Ceramide, (**B**) Hexosyl-Ceramide, and (**C**) Sphingomyelin. Data presented as mole percent composition of each species (mean ± S.D.). WT, wild-type; KO, *Sphk2* knockout. *p < 0.05; **p < 0.01.

We analyzed the plasma, liver and retina from *Sphk2* KO and WT littermate mice for the major sphingolipids (ceramide, Cer; hexosyl-ceramide, Hex-Cer; sphingomyelin, SM) and long chain base containing species such as Sphingosine (Sph), dihydro sphingosine (DH Sph), sphingosine 1-phosphate (S1P), and dihydro sphingosine 1-phosphate (DHS1P) by mass spectrometry. In plasma, we observed significant increases in C24:1 Cer (p < 0.05, n = 4), and both C16:0 and C22:0 Hex-Cers (p < 0.01, n = 4) in *Sphk2* KO mice (Fig. 4A,B). In the liver, we detected very significant increases of C16:0 Cer (p < 0.001, n = 4) and C16:0 Hex-Cer (p < 0.05, n = 4), along with decreases in C24:0 Cer (p < 0.05, n = 4) and C22:0 Hex-Cer (p < 0.01, n = 4) (Supplementary Fig. S4). In retinal tissue, however, we only found a significant increase in C18:0 Cer in *Sphk2* KO mice (Supplementary Fig. S5; p < 0.05, n = 4, t-test). Knocking out or blocking SPHK2 is known to increase the levels of S1P in the plasma and blood^32,33^, which might have affected or shifted the sphingolipid metabolism towards the observed profile in the above tissues. No changes were observed in any species of SM (Supplementary Figs S4, S5) and these profiles were not changed after 3 hours of systemic treatment with FTY720 at 10 mg/kg (data not shown).

**Figure 5:**
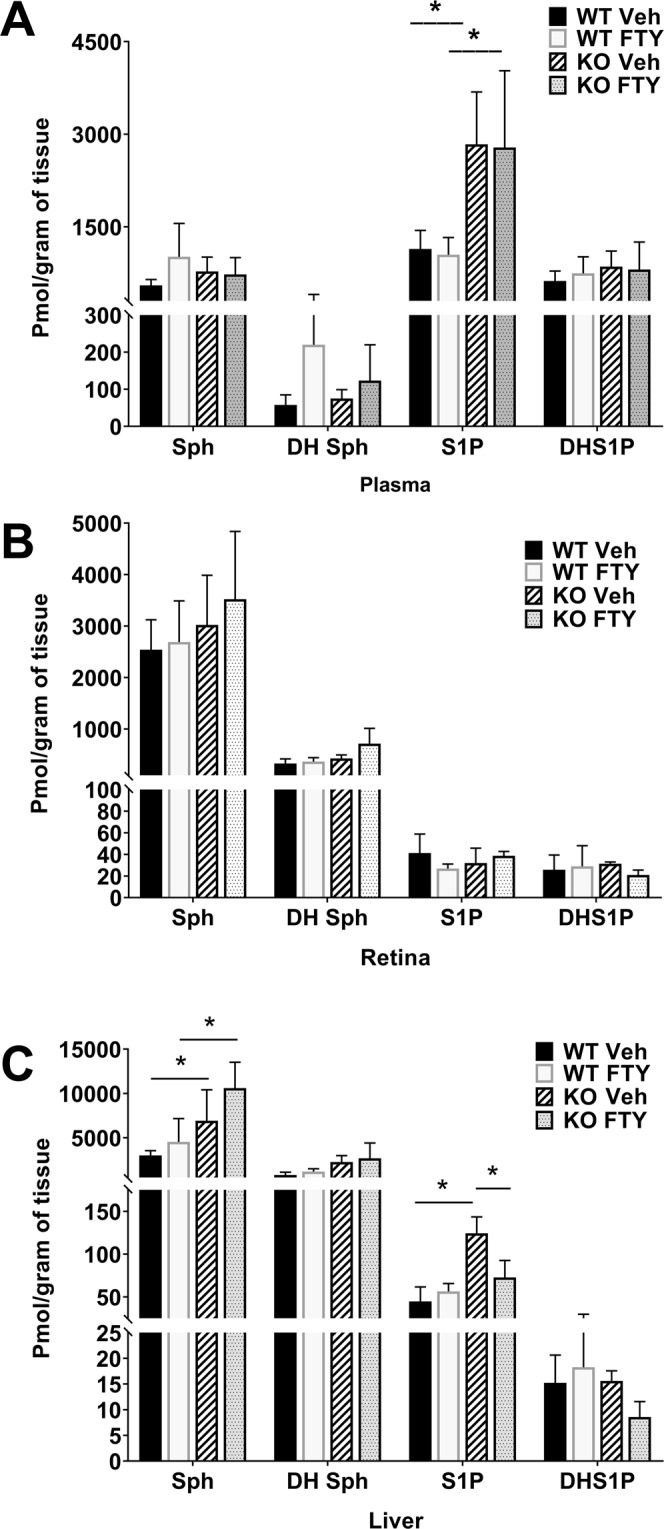
Long Chain Base species in *Sphk2* knockout mice with and without FTY720. Samples from (**A**) Plasma, (**B**) Retina, and (**C**) Livers were collected from WT and *Sphk2* KO mice 3 hours after systemic delivery of FTY720 at 10 mg/kg and analyzed for various species of long-chain sphingolipid bases using LC/MS/MS and presented as pmol per gram of tissue sample (mean ± S.D). WT, wild-type; KO, *Sphk2* knockout; Veh, Vehicle; FTY, FTY720; Sph, Sphingosine; DH Sph, dihydrosphingosine; S1P, Sphingosine 1-Phosphate; DH S1P, dihydrosphingosine 1-Phosphate. *p < 0.05.

We further confirmed that knocking out of *Sphk2* increases the levels of S1P in the plasma. As shown in Fig. 5A, we detected a > 2-fold increase in S1P levels in the plasma of KO mice (p < 0.05, n = 6; two-way ANOVA with Bonferroni’s correction). No changes were found in other long-chain sphingosine base (LCB) containing species (Fig. 5A). We also did not find any significant effect of FTY720 treatment on the other LCB levels in either WT or KO mice (Fig. 5A). In the retina, no difference in the LCBs were found due to deletion of *Sphk2* or treatment with FTY720 (Fig. 5B). In the liver however, deletion of *Sphk2* was shown to increase S1P levels as well as Sph levels (Fig. 5C; p < 0.05, n = 6; two-way ANOVA with Bonferroni’s correction). However, when these KO mice were treated with FTY720, they had reduced S1P levels in the liver and significant Sph accumulation (Fig. 5C). Therefore, it appears that deactivation of SPHK2, which is the primary form of sphingosine kinase in the liver, slowed or reduced phosphorylation of Sph, causing an accumulation of Sph (in the order of thousandth, where S1P increase is hundredth pmol, which is further increased by FTY720 treatment as FTY720 competes with Sph as substrate for the SPHK enzymes (Fig. 5C).

### Metabolism and bioavailability of FTY720 in *Sphk2 KO* retina and other tissues

**Figure 6:**
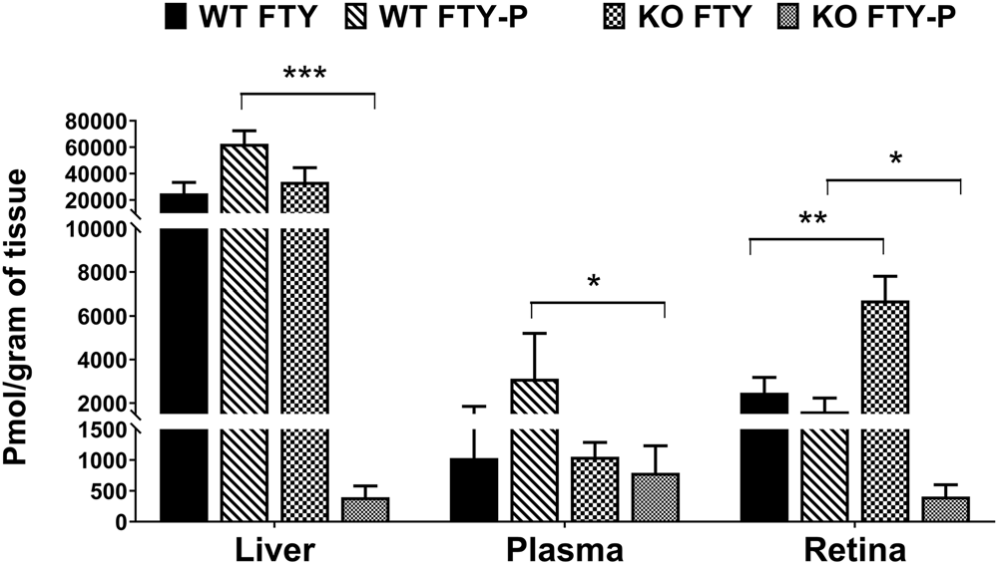
Bioavaility and metabolism of FTY720 in *Sphk2* knockout and wild-type mice. Liver, plasma, and retina samples were collected from WT and *Sphk2* KO mice 3 hours after FTY720 injection and analyzed for FTY720 and FTY720-P quantification using LC/MS/MS and presented as pmol per gram of tissue sample (mean ± S.D.). WT, wild-type; KO, knockout; FTY, FTY720; FTY-P, FTY720-Phosphate. *p < 0.05; **p < 0.01; ***p < 0.001.

There is no report on FTY720 bioavailability and metabolism in the retina. In this case, we wanted to investigate the conversion and presence of FTY720-P in the retina during light exposure. We therefore measured the absolute levels (pmol/g tissue) of FTY720 and FTY720-P in liver, plasma, and retinal samples from KO and WT mice at 3 hours following FTY720 injection. We observed no difference in FTY720 levels in the liver and plasma of the KO mice when compared to the WT mice (Fig. 6; p < 0.05, n = 6; two-way ANOVA). Retinal levels of FTY720 were found to be significantly higher in KO animals at 3 hours post-injection (Fig. 6; p < 0.01, n = 3; two-way ANOVA). The levels of FTY720-P were significantly decreased in all KO mice tissues, illustrating that SPHK2 is responsible for the majority of FTY720 conversion (Fig. 6; p < 0.05). Plasma FTY720-P was decreased but not to the extent of liver and retina in KO mice, suggesting that plasma SPHK1 was activated to compensate SPHK2 deletion and thus increased the S1P levels in the plasma (Fig. 5A) and similarly converts FTY720 to FTY720-P.

**Figure 7:**
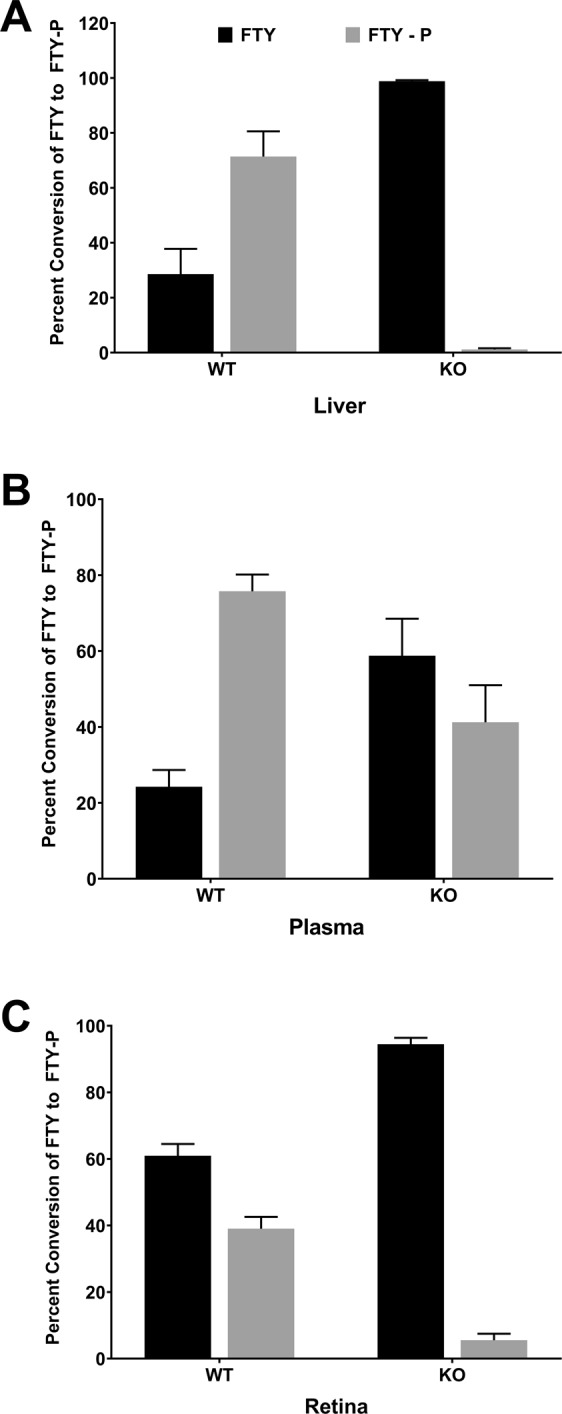
FTY720 and FTY720-P ratio in various tissues of *Sphk2* knockout and wild-type mice. The percentage of FTY720 relative to FTY720-Phosphate was measured by LC/MS/MS in both WT and *Sphk2* KO mice tissue (mean ± S.D.): (**A**) liver, (**B**) plasma, and (**C**) retinas. WT, wild-type; KO, *Sphk2* knockout; FTY, FTY720; FTY-P, FTY720-Phosphate.

In order to understand the extent of conversion of FTY720 to FTY720-P and their relative ratio in different tissues, we determined percent ratios of FTY720 to FTY720-P in liver, plasma, and retinal tissue from WT and KO mice. In WT livers 3 hours post-treatment, the ratio of FTY720 to FTY720-P was 30:70, whereas KO mice injected with FTY720 were found to have almost no FTY720-P in liver samples, indicating that inactivation of Sphk2 almost completely prevented phosphorylation of FTY720 in the liver (Fig. 7A; n = 6). The plasma samples of KO mice showed markedly decreased FTY720 conversion, though not as profoundly as in the liver. The ratio of FTY720 to FTY720-P was 23:77 in WT plasma and 58:42 in KO plasma, respectively (Fig. 7B; n = 6). As mentioned earlier, this is most likely due to compensatory activity of Sphk1, which is a secretory enzyme and found in high concentration in blood and in erythrocytes^34–37^. SPHK1 has lower conversion efficiency for FTY720 than SPHK2, but it is possible that the inefficiency was offset by the high levels of FTY720 substrate. WT retinas were found to be less efficient in phosphorylating FTY720 than the liver and the plasma, as we detected the ratio of FTY720 to FTY720-P to be 60:40 after 3 hours (Fig. 7C; n = 3), whereas it was 30:70 and 23:77 in livers and plasma, respectively. It may be possible that expression/activity of *Sphk2* in retinal tissue is lower than in liver and plasma. However, phosphorylation of FTY720 decreased significantly in KO retinas, with a ratio of 96:4 (Fig. 7C; n = 3). Our data clearly shows that FTY720-P levels are minimal in *Sphk2* KO retina. It also suggests that SPHK1 has minimal influence on the amount of FTY720-P reaching the retina. Thus, FTY720 appears to be the active compound preventing photoreceptor degeneration due to light damage and we rule out the effect of FTY720-P in doing so.

## Discussion

Sphingolipids are vital in maintaining the viability of neuronal tissue. Through cycles of degradation and re-synthesis of different sphingolipid metabolites, sphingolipids can regulate pathways for proliferation, apoptosis, angiogenesis, and migration, and sphingolipid signaling is potentially involved in a variety of mammalian retinal degenerative diseases^22,23,38^. Our prior findings have implicated systemic FTY720, a Sph analogue and FDA-approved drug, in protection from light- and disease-mediated retinal degeneration^22^. These findings underscore the potential for development of novel therapeutics which target sphingolipid signaling and metabolic pathways. However, it is necessary to first develop a detailed understanding of FTY720’s metabolism, pleiotropic biological actions, and the mechanisms by which it or its phosphorylated form prevents retinal degeneration. From our previous studies, we hypothesized that FTY720 that prevents retinal degeneration by inhibiting *de novo* Cer synthesis^22,23,38^. In the present study, we used *Sphk2* KO mice to determine if FTY720 requires phosphorylation to FTY720-P prior to assuming its protective role in retinal degeneration.

FTY720 is a sphingosine analog which is phosphorylated in mammals to FTY720-P and functions as an agonist for four of the five S1P receptors (1, 3, 4 and 5)^39,40^. FTY720 is also an FDA-approved drug for MS; the phosphorylated form of FTY720 is able to sequester circulating lymphocytes, causing lymphopenia^39–42^. Independent actions of FTY720 have recently been demonstrated in many cellular activities^29^. For instance, FTY720 inhibits the cannabinoid CB1 receptor^43^ and cytosolic phospholipase A2 (cPLA2), and counteracts ceramide 1-phosphate-induced cPLA2 activation^44^. FTY720 is also known to inhibit S1P lyase, another important enzyme for cellular sphingolipid metabolism and regulation^45^. Many studies have proven that hepatic SPHK2 is the primary enzyme which phosphorylates FTY720 to FTY720-P, which is assumed to be the biologically active form of the drug, at least in the context of MS^2,15–19^. We previously acquired compelling evidence that lymphopenia is not the cause of FTY720-induced retinal protection from light-induced damage^22^. As a result of light-induced damage, the retinal photoreceptors degenerate and undergo apoptosis, but the apoptotic cascade can be blocked by FTY720. FTY720’s action is complex and poorly-understood in the context of retinal protection: the drug is phosphorylated to FTY720-P, which acts as an S1P mimic, and S1P is active in a wide range of cellular processes including growth, differentiation, motility, calcium mobilization, and other processes which are conducive to cell survival^14,46^. S1P is also important for the development of the vascular system^6,9–11^, the heart^47^, and for immunity^5,7,12^. The S1P signaling pathway through S1P receptors can induce activation of the small GTPase Rac, phospholipase C, extracellular signal-regulated kinase, protein kinase Akt, phosphatidylinositol 3-kinase, etc.^8,13^. It is therefore important to understand the extent of FTY720-P’s involvement, if any, in retinal protection from apoptosis.

FTY720 is primarily phosphorylated in the liver by SPHK2^15–19^. We therefore used *Sphk2* KO mice generated by Proia group which are severely deficient in their ability to phosphorylate FTY720^13^. This line of *Sphk2* KO mice, and two other lines developed independently, have no phenotype due to knocking out the *Sphk2* gene^13,16,20^. We conducted basic visual functional characterization of these mice and did not find any major differences from their WT littermates until 6 months of age (Fig. 1). We found their vulnerability to light-induced damage to be comparable to WT mice. The KO mice have little resistance to damage as evidenced by structural data, but functional deterioration was similar to WT mice. We also found that both *Sphk2* KO mice and littermate WT controls are similarly protected from damaging light by FTY720 treatment (Figs 2, 3).

We also determined the bioavailability and metabolism of FTY720 in the retina, liver, and plasma. In *Sphk2* KO mice, 3 hours following FTY720 delivery, almost no FTY720-P was found in liver samples, whereas almost 80% of FTY720 was converted to FTY720-P in WT livers, indicating that inactivation of SPHK2 almost completely prevented hepatic phosphorylation of FTY720 (Figs 6, 7). Plasma levels of both FTY720 and FTY720-P were much lower than in the liver (~20 fold) and showed markedly decreased FTY720 conversion in KO samples, though not as profoundly as in the liver (Figs 5, 7). This is likely due to SPHK1, which is present in the plasma and found in higher concentrations in erythrocytes^34–37^. KO retinas had significantly decreased FTY720-P, indicating that the liver is the major source for FTY720-P bound for the retina (Figs 6, 7). The retina has very low expression of both *Sphk1* and *Sphk2* genes^48^ and retinal levels of S1P are 30–40-fold lower than plasma levels (Fig. 5B). From mouse and human tissue distribution studies, SPHK2 is expressed mostly in the brain, kidney, liver, lungs, spleen and blood^16,20,21^. Our data suggests that SPHK1 has minimal influence in the retina under the *Sphk2* KO background. However, there is a chance that circulating plasma FTY720-P may have influenced retinal levels to a minor extent. The retina has a small amount of vasculature which may allow for transfer of S1P and FTY720-P from the plasma. However, we did not see a change in retinal S1P levels in *Sphk2* KO mice despite major changes in plasma S1P levels, suggesting that this transfer is limited (Fig. 5). We have previously noted changes in retinal S1P levels following light-induced damage^22^ and our data suggests that FTY720 has higher bioavailability in the retina than FTY720-P (Figs 6, 7). Furthermore, FTY720-P levels were very low (<5%, Fig. 7) in *Sphk2* KO retinas. Our previous studies suggest that FTY720 protection of the retina is dose-dependent^22^, so the likelihood of a significant protective effect attributable to the very low levels of FTY720-P present in *Sphk2* KO retinas is minimal. Whole retinas were used for measurements of sphingolipid and FTY720 levels, meaning that these measurements are not fully specific to photoreceptors. However, as a very large majority of the murine retina is composed of photoreceptor cells^49^, these measurements should closely reflect actual levels of sphingolipids, FTY720, and FTY720-P specific to the photoreceptor cells. Our data therefore strongly suggests that FTY720, rather than FTY720-P, is active in retinal cell protection. They also suggest a potential direct action of FTY720 in the modulation of sphingolipid (Ceramide) signaling in the retina and protection of retinal cells from apoptotic death.

In conclusion, this is the first retinal characterization of *Sphk2* KO mice, which are structurally and functionally similar to WT mice of similar background. As expected, these mice are deficient in FTY720 phosphorylation; however, extreme reduction of FTY720-P did not affect retinal protection associated with systemic FTY720 dosage. This observation supports the hypothesis that FTY720 can protect the mammalian retina from apoptotic cell death without phosphorylation. This study is therefore a step towards identifying novel targets in the sphingolipid metabolic pathways for human retinal degenerative diseases causing photoreceptor cell apoptosis, and a step towards potentially developing treatments utilizing FTY720 or related compounds.

## Materials and Methods

### Animal care

All procedures were performed according to the Association for Research in Vision and Ophthalmology Statement for the Use of Animals in Ophthalmic and Vision Research and the University of Oklahoma Health Sciences Center (OUHSC) and University of Tennessee Health Science Center (UTHSC) Guidelines for Animals in Research. Wild-type (WT) and *Sphk2* global knockout (KO) mice (BALB/c background) were generated from pigmented mice that were received from Dr. Richard L. Proia (NIDDK, Bethesda, MD). The mice were born and raised in the Dean A. McGee Eye Institute vivarium at OUHSC and maintained from birth under dim cyclic light (5–10 lux, 12 hours on/off, 7 a.m. to 7 p.m. CST). All mice were genotyped for the retinal degeneration mutations, rd1 and rd8, to ensure they were not present. All procedures, tissue harvest and the methods of euthanasia for mice were reviewed and approved by the OUHSC and UTHSC Institutional Animal Care and Use Committee. Mice were euthanized by carbon dioxide asphyxiation before harvesting the eye or retinal tissues.

### Retinal light damage and FTY720 treatment

Light damage was induced by exposing albino *Sphk2* KO mice and their WT littermates to damaging white light at an intensity of 1000 lux for 10 hours at night when the mice are alert and active (6 PM to 4 AM). Following light damage, the mice were returned to their normal room for 7 days. After 7 days, visual function was tested by Electroretinography (ERG). Mice were then euthanized via CO2 asphyxiation and their eyes were then harvested for histological analyses.

To determine the protective role of FTY720, FTY720 (Selleck Chemicals; Houston, TX) was dissolved in a vehicle containing sterile DMSO (100%) (Sigma; St Louis, MO) and saline (0.9%) 1:4 (v/v). Mice were treated with a single dose of FTY720 at 10 mg/kg total body weight by IP injection 0.5 hours prior to the start of LD. The vehicle group received only DMSO and saline (1:4) vehicle. 7 days after light damage, the mice were tested using ERG and their eyes were harvested for histology.

### Electroretinography (ERG)

Flash ERGs were recorded for both eyes with the Diagnosys Espion E2 ERG system (Diagnosys LLC, Lowell, MA) following previously-published protocols^22,23,30^. For the assessment of rod photoreceptor function (scotopic ERG), five strobe flash stimuli were presented at flash intensities of 0.0004, 0.04, 4, 400, and 2000 candela (cd)·s/m^2^. The amplitude of the A-wave was measured from the pre-stimulus baseline to the A-wave trough. Scotopic B-wave response was measured for secondary neurons. The amplitude of the B-wave was measured from the trough of the A-wave to the peak of the B-wave. For the evaluation of cone photoreceptor function (photopic ERG), a strobe flash stimulus (2000 cd·s/m^2^) was presented to dilated, light-adapted (5 minutes at 100 cd·s/m^2^) mice at 3 and 6 months of age. Cone functional analysis used various flash intensities and frequencies (Photopic 2000, Green, Blue, 3 Hz, 10 Hz, 20 Hz, 30 Hz), under a steady adapting field of 1.7 log cd.s/m^2^. The amplitude of the cone B-wave was measured from the trough of the A-wave to the peak of the B-wave. Functional characterization was recorded for WT and *Sphk2* KO mice at 3 and 6 months of age and seven days after light-induced retinal damage with or without injections of FTY720 or vehicle.

### Histology

After ERG recordings, mice were euthanized by carbon dioxide asphyxiation and one or both mouse eyes were enucleated immediately, marked for orientation, placed in fixative (Prefer; Anatech LTD, Battle Creek, MI), and embedded in paraffin for light microscope evaluation of retinal structure. Sections of 5 μm were cut along the vertical meridian through the optic nerve and stained with hematoxylin and eosin (H & E). The thickness of the outer nuclear layer (ONL) was measured along the vertical meridian every 0.2–0.3 μm of the retina starting from the optic nerve head (ONH) and moving out to the inferior and superior ora serrata. Measured points were plotted as a spider diagram in GraphPad Prism 7.03. ONL thickness was measured for *Sphk2* KO and WT mouse retinas at 6 months and for KO and WT mice with or without FTY720 or vehicle injection seven days after light-induced retinal damage.

### Extraction and analysis of sphingolipids and FTY720 and FTY720-P

WT and *Sphk2* KO animals were treated with 10 mg/kg of FTY720 by IP injection and tissues such as retina, liver, and plasma were harvested after 3 hours. Plasma was also collected to generate SPL profiles of Cer, HexCer, and SM for WT and *Sphk2* KO mice without FTY720 injection. Harvested tissues were snap frozen in liquid nitrogen and used for sphingolipid, FTY720, and FTY720-P analyses. Sphingolipids, FTY720, and FTY720-P were analyzed by LC/MS/MS at the Lipidomic Core Facility at Virginia Commonwealth University. We have previously published the procedures for sphingolipid analysis^23,50^. FTY720 and FTY720-P were also extracted with sphingolipids and integrated with sphingolipid internal standards. FTY720 and FTY720-P were measured in lipid extracts by LC-ESI-MS/MS with the Sciex 5500 QTRAP. Briefly, FTY720 and FTY720-P were analyzed in the same LC-ESI-MS/MS run as sphingolipid bases and sphingoid base 1-phosphates in the positive ion mode with multiple reaction monitoring transitions of m/z 308.0-255.2 and m/z 388.2-255.2 respectively. FTY720 was shown to extract with a similar efficiency to d17:1 Sph, and FTY720-P was shown to extract similarly to d17:1 S1P. To allow for quantitation, standard curves were generated at 0.5 pmol to 1.0 nmol on column for d17:1 Sph, d17:1 S1P, FTY720, and FTY720-P. Based on the relative ionization efficiencies between these curves, conversion factors were applied to areas for the traditional d17:1 Sph and d17:1 S1P internal standards, thus allowing the quantitation of FTY720 and FTY720-P without addition of further internal standards.

### Statistics

All statistics were done using GraphPad Prism version 7.03 for Windows (GraphPad Software, La Jolla, CA). Column data were assessed for normality to determine whether to use two-tailed t-tests or Mann-Whitney two-tailed tests (α = 0.05). Grouped data was evaluated by two-way ANOVA (α = 0.05) followed by Bonferroni’s correction for multiple comparisons. For all the data presented, n represents the number of animals. For ERG and histology, data from both eyes were averaged and mean and S.D. were determined from the number of animals.

## Supplementary information

Supplementary Information

## Data Availability

The datasets generated during and/or analyzed during the current study are available from the corresponding author on reasonable request.
