# Supplementary material for: Sphingosine Kinase 2 Phosphorylation of FTY720 is Unnecessary for Prevention of Light-Induced Retinal Damage
Source: Sci Rep. 2019 May 23;9:7771. doi: 10.1038/s41598-019-44047-z (PMC6533254; doi:10.1038/s41598-019-44047-z)
Supplement: Supplementary file 1 — Supplementary Information [file 41598_2019_44047_MOESM1_ESM.pdf]

Sphingosine Kinase 2 Phosphorylation of FTY720 is Unnecessary for Prevention of Light-Induced Retinal Damage

Hui Qi[1], Jerome Cole II[2], Richard C Grambergs[2], John R Gillenwater[2], Koushik Mondal[2], Sufiya Khanam[2], Soma Dutta[2], Megan Stiles[1], Richard L. Proia[3], Jeremy Allegood[4], Nawajes Mandal[1,2,5]

SUPPLEMENTARY FIGURES:

Supplementary Figure S1

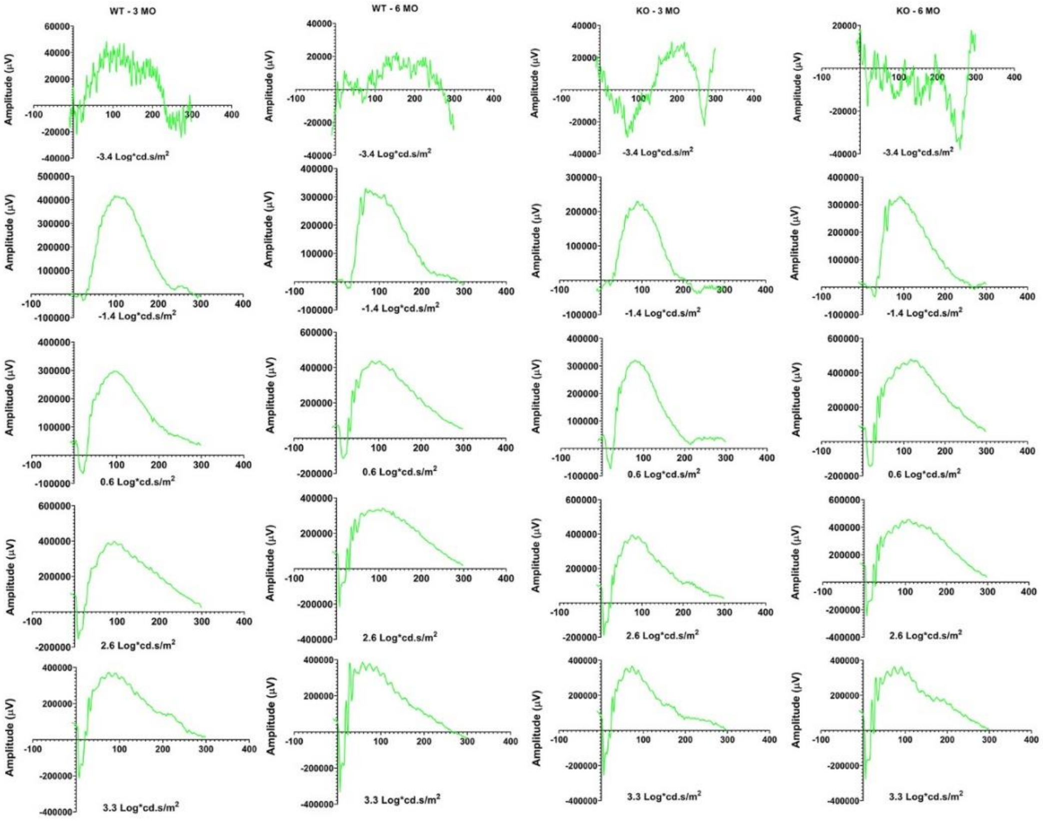

Supplementary Figure S1: **Electroretinography (ERG) representative traces.** Figure shows representative traces of wild-type (WT) and Sphingosine Kinase 2 knockout (*Sphk2* KO) mice at 3 and 6 months of age. Traces show similar photoreceptor viability between WT and KO mice at 3 and 6 months as indicated by similar A-wave and B-wave amplitudes (µV) at various flash intensities (-3.4, -1.4, 0.6, 2.6, and 3.3 Log*cd.s/m^2). The absence of the SK2 gene does not cause any apparent defects or functional abnormalities in photoreceptor performance.

Supplementary Figure S2

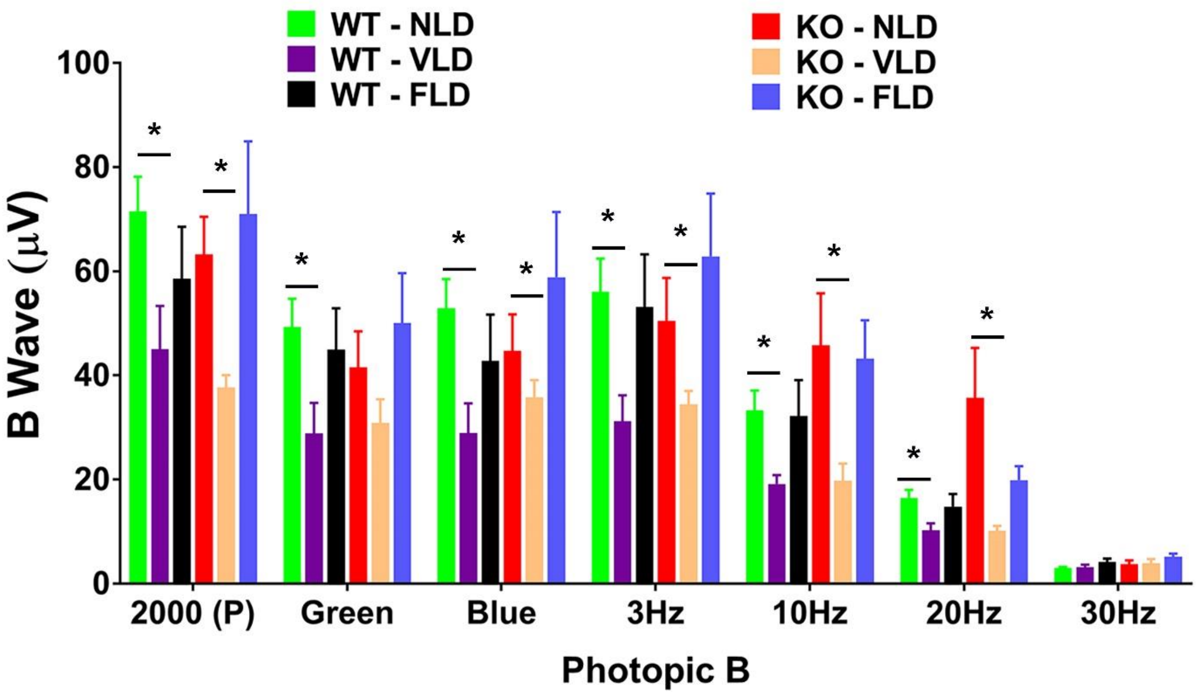

Supplementary Figure S2. **Photopic ERG chracterization of *Sphk2* KO mouse retina after light damage.** Seven days after light damage, cone photoreceptor function of WT and *Sphk2* KO mice was measured by light-adapted ERG at various flash intensities and frequencies (Photopic 2000, Green, Blue, 3Hz, 10Hz, 20Hz, 30Hz), under a steady adapting field of 1.7 log cd.s/m^2. Mice were either injected with FTY720 or Vehicle, or received no light damage. B-wave amplitude is presented as mean ± S.D. WT-NLD, n = 24; WT-VLD, n = 12; WT-FLD, n = 12; KO-NLD, n = 18; KO-VLD, n = 12; KO-FLD, n = 16). WT, wild-type; KO, knockout; NLD, No Light Damage; VLD, Vehicle Light Damage; FLD, FTY720 Light Damage. *p<0.05.

Supplementary Figure S3

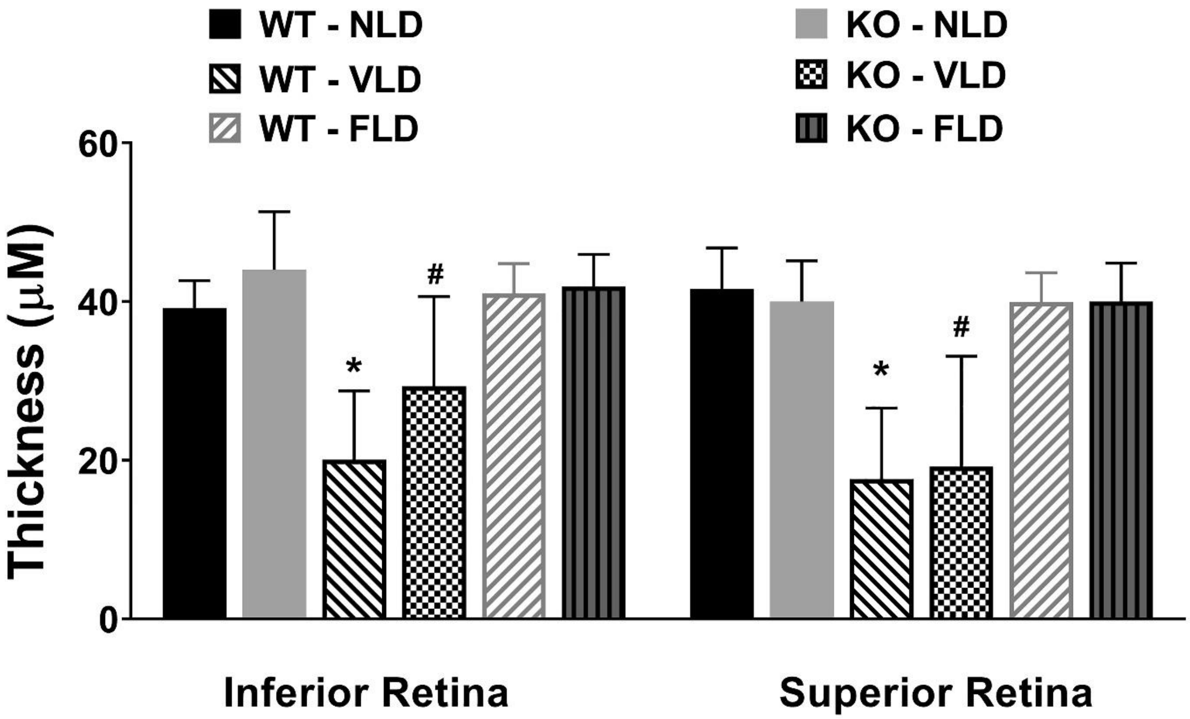

Supplementary Figure S3: **Averaged thickness of the Outer Nuclear Layer (ONL) at the superior and inferior central retina of wild-type (WT) and Sphingosine Kinase 2 knockout (*Sphk2* KO) mice.** Mice were subjected to light-induced retinal damage with intraperitoneal FTY720 injection (FLD), vehicle injection (VLD), or no light damage and no injection (NLD). Retinal thickness at 4 points on each side was measured and presented as mean ± S.D. Inferior retina: WT-NLD, n = 3; KO-NLD, n = 3; WT-VLD, n = 10; KO-VLD, n = 7; WT-FLD, n = 10; KO-FLD, n = 19; Superior retina: WT-NLD, n = 3; KO-NLD, n = 3, WT-VLD, n = 10; KO-VLD, n = 7; WT-FLD, n = 10; KO-FLD, n = 19. *$p<0.05$ (WT-NLD vs. WT-VLD) and $^{\#}p<0.05$ (KO-NLD vs. KO-VLD).

Supplementary Figure S4

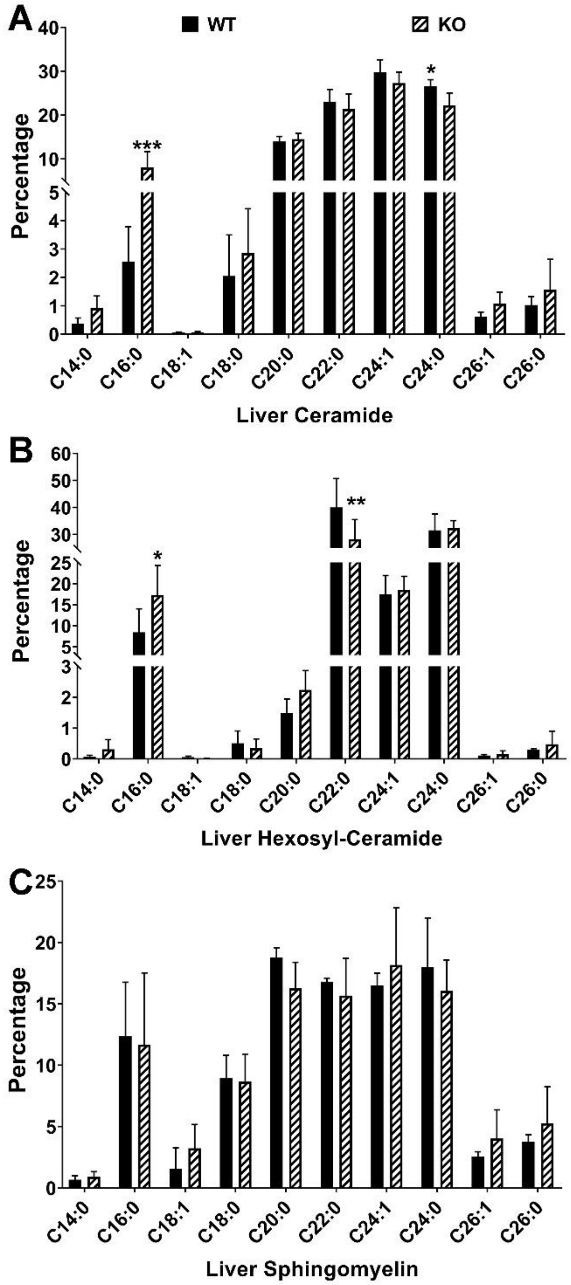

Supplementary Figure S4. **Sphingolipid Species of *Sphk2* KO mice in Liver.** Liver tissue samples were collected from WT and *Sphk2* KO mice and analyzed using LC/MS/MS for relative levels of various chain length variants of the major sphingolipid classes: **A)** Ceramide, **B)** Hexosyl-Ceramide, and **C)** Sphingomyelin. Data presented as mole percent composition of each species (mean ± S.D.; n = 4). WT, wild-type; KO, knockout; *p<0.05; **p<0.01; ***p<0.001.

Supplementary Figure S5

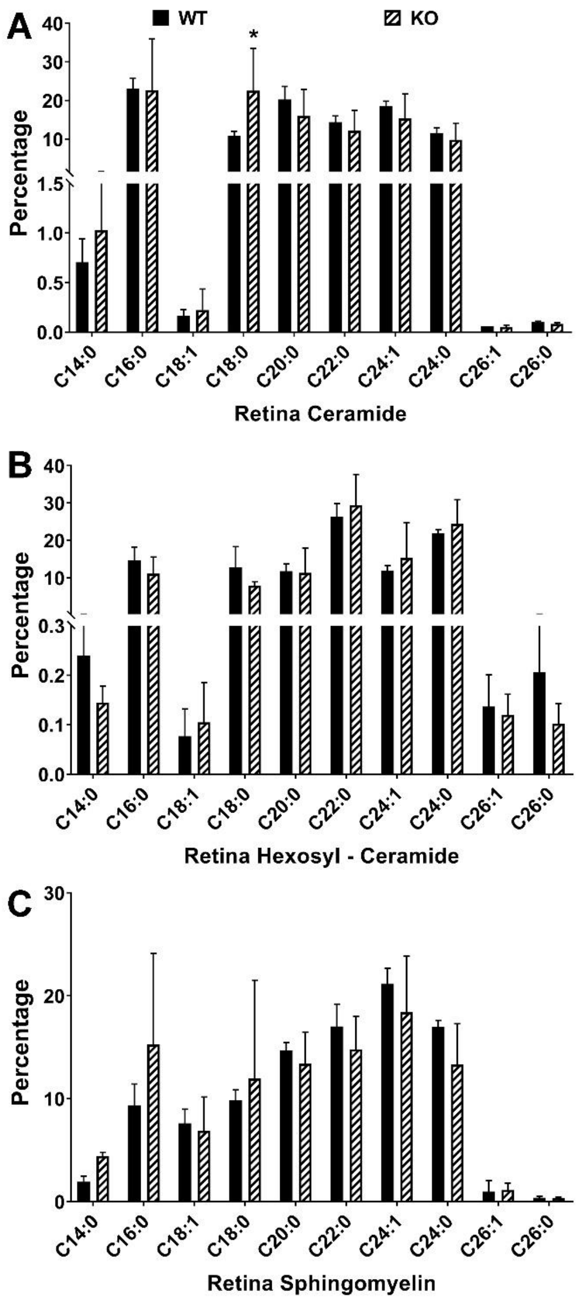

Supplementary Figure S5. **Sphingolipid Specie of *Sphk2* KO mice in Retina.** Retina tissue samples were collected from WT and *Sphk2* KO mice and analyzed using LC/MS/MS for relative levels of various chain length variants of the major sphingolipid classes: **A)** Ceramide, **B)** Hexosyl-Ceramide, and **C)** Sphingomyelin. Data presented as mole percent composition of each species (mean ± S.D.; n = 4). WT, wild-type; KO, knockout; *$p<0.05$.
